# Experience dependent plasticity of higher visual cortical areas in the mouse

**DOI:** 10.1093/cercor/bhad203

**Published:** 2023-06-03

**Authors:** Rosie Craddock, Asta Vasalauskaite, Adam Ranson, Frank Sengpiel

**Affiliations:** School of Biosciences, Cardiff CF10 3AX, UK; Neurosciences and Mental Health Research Institute, Cardiff University, Cardiff CF10 3AT, UK; School of Biosciences, Cardiff CF10 3AX, UK; Neurosciences and Mental Health Research Institute, Cardiff University, Cardiff CF10 3AT, UK; Faculty of Medicine and Health Sciences Department of Basic Sciences, Universitat Internacional de Catalunya, Barcelona 08195, Spain; Institut de Neurociènces, Universitat Autònoma de Barcelona, Bellaterra 08193, Spain; School of Biosciences, Cardiff CF10 3AX, UK; Neurosciences and Mental Health Research Institute, Cardiff University, Cardiff CF10 3AT, UK

**Keywords:** ocular dominance, orientation selectivity, two-photon calcium imaging, visual cortex

## Abstract

Experience dependent plasticity in the visual cortex is a key paradigm for the study of mechanisms underpinning learning and memory. Despite this, studies involving manipulating visual experience have largely been limited to the primary visual cortex, V1, across various species. Here we investigated the effects of monocular deprivation (MD) on the ocular dominance (OD) and orientation selectivity of neurons in four visual cortical areas in the mouse: the binocular zone of V1 (V1b), the putative “ventral stream” area LM and the putative “dorsal stream” areas AL and PM. We employed two-photon calcium imaging to record neuronal responses in young adult mice before MD, immediately after MD, and following binocular recovery. OD shifts following MD were greatest in LM and smallest in AL and PM; in LM and AL, these shifts were mediated primarily through a reduction of deprived-eye responses, in V1b and LM through an increase in response through the non-deprived eye. The OD index recovered to pre-MD levels within 2 weeks in V1 only. MD caused a reduction in orientation selectivity of deprived-eye responses in V1b and LM only. Our results suggest that changes in OD in higher visual areas are not uniformly inherited from V1.

## Introduction

Since the pioneering work by Hubel and Wiesel on cats and monkeys in the 1960s and 1970s (Wiesel and Hubel 1963; Hubel et al. 1977) ocular dominance (OD) plasticity has become one of the classic paradigms for the study of experience-dependent brain plasticity. Occluding one eye by monocular deprivation (MD) shifts responses of visual cortical neurons toward the open eye, in particular during a critical period early in life (Hubel and Wiesel 1970; Olson and Freeman 1978). It also results in a substantial loss of visual acuity in the deprived eye (Dews and Wiesel 1970; Harwerth et al. 1983), a condition known as amblyopia. Studies of OD plasticity have for over 50 years focused on the primary visual cortex (V1), although it is accepted that not all visual deficits observed in amblyopic animals can be accounted for by the abnormalities of responses seen in V1 (see Kiorpes and Daw 2018).

Although neurons in the mouse primary visual cortex, unlike most primate and carnivore species, are not organized into readily distinguishable OD columns (but see Goltstein et al. 2022) and are overall more strongly dominated by the contralateral eye (Dräger 1975), they nevertheless respond to MD with a characteristic OD shift toward the open eye, as originally demonstrated in single-cell recording studies (Dräger 1978; Gordon and Stryker 1996) and later confirmed using two-photon imaging (Mrsic-Flogel et al. 2007; Rose et al. 2016). Mouse V1 is most susceptible to the effects of MD during the critical period, peaking at P28 (Gordon and Stryker 1996), but OD shifts can still be observed in older animals (Sawtell et al. 2003), and in some cases, even in animals up to at least 8 months of age, depending on rearing conditions (Greifzu et al. 2014). From a number of studies a consensus has emerged that OD plasticity in V1 of young mice within the classical critical period is mechanistically different from that observed in animals that are two months of age or older (for reviews, see Espinosa and Stryker 2012; Levelt and Hübener 2012). During the critical period, a brief MD of 3 days results in a Hebbian, LTD-type depression of deprived-eye responses (Bear et al. 1990), whereas slightly longer MD of 6 days is characterized by a homeostatic upregulation of both the open-eye and (to a lesser extent) the deprived-eye responses, which requires tumor necrosis factor-alpha (Kaneko et al. 2008b). Upon re-opening of the deprived eye responses through both eyes return to baseline levels, and this recovery is dependent upon brain-derived neurotrophic factor via tyrosine kinase B receptor activation (Kaneko et al. 2008a). In contrast, OD plasticity in (young) adult mice under standard rearing conditions is apparent only after 6–7 days of MD and is typically characterized by an increase in the open-eye response, with little change in the deprived-eye response, and this is NMDA receptor-dependent (Sawtell et al. 2003; Ranson et al. 2012).

Despite the wealth of knowledge concerning OD plasticity in the primary visual cortex of mice very little is known about higher visual areas (HVAs) in this respect, at any age; this may be explained by the fact that in the past most studies were carried out on anesthetized animals and anesthesia would likely have affected HVA responses. Recent studies have revealed up to 15 retinotopically organized HVAs surrounding V1 (Wang and Burkhalter 2007; Garrett et al. 2014; Zhuang et al. 2017), and it has been suggested on the basis of axonal projection patterns that they are organized into to two groups, which may be equivalent to the dorsal and ventral processing streams known from the primate visual system (Wang et al. 2011; Wang et al. 2012). Despite some anatomical crosstalk between the two streams they appear to be distinct in terms of their spatiotemporal selectivity (Murakami et al. 2017), although the high spatial frequency selectivity of PM appears at odds with it being assigned to the dorsal stream (Andermann et al. 2011; Marshel et al. 2011).

Here we explore OD plasticity in higher visual areas that are direct downstream targets of binocular V1, including areas both in the putative dorsal stream (AL, PM) and ventral stream (LM). We specifically aim to address the questions whether the higher visual areas simply inherit plastic changes occurring in V1 or independently express experience-dependent plasticity, and whether dorsal and ventral streams exhibit different forms or degrees of plasticity. We use two-photon calcium imaging of awake animals to analyze the effects of MD in young adult mice on responses of individual neurons and subsequent recovery under binocular vision.

## Materials and methods

### Animals

All experimental procedures were carried out in accordance with the UK Animals (Scientific Procedures) Act 1986 and European Commission directive 2010/63/EU. Mice expressing GCaMP6f in excitatory neurons were generated by crossing the Ai95D line (Jax, 024105) with the CaMkII-alpha-cre T29–1 line (Jax, 005359). Experiments were carried out on young adult mice of either gender aged 2–3 months. Same sex littermates were housed together under normal light conditions (12-hour light, 12-hour dark), and environmental enrichment was provided in form of nesting material, a cardboard, and a Perspex tube and a chewstick. All recordings were made during the light period.

### Surgery

Surgical procedures were in line with recently published recommendations (Barkus et al. 2022). All surgical procedures were conducted under aseptic conditions.

Mice were monocularly deprived by eyelid suture under isoflurane anesthesia (2% in O_2_, 0.6 L/min). Lid margins were trimmed, and the eyelids were sutured with three mattress stitches. Eyelids were reopened under anesthesia 2 weeks later. The integrity of the deprivation was checked daily; the experiment was discontinued if deprivation was impaired or if after re-opening corneal opacities were detected.

**Fig. 1:**
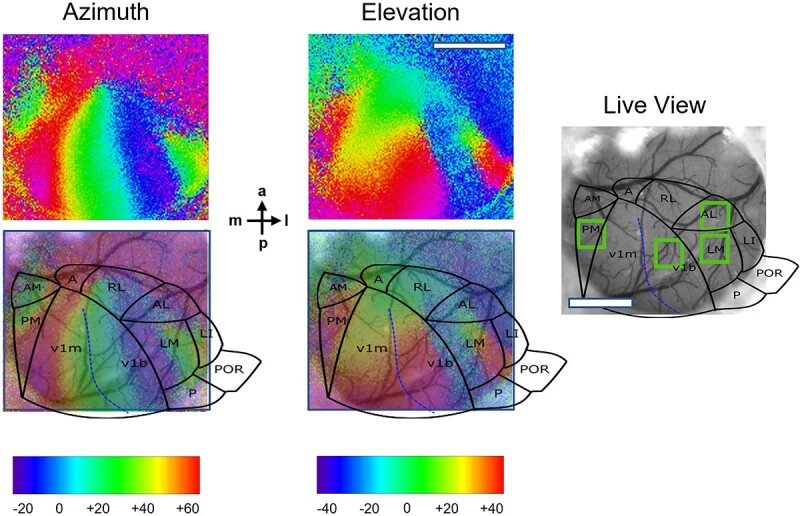
Identification of V1b, LM, AL, and PM based on retinotopic mapping of the mouse visual cortex. Drifting vertical and horizontal bars were shown separately to the left (contralateral) and right (ipsilateral) eye using a periodic stimulation protocol (for details, see methods). The stimulus phases producing the maximal response are color coded and represent retinotopic azimuth and elevation, respectively. Each visual cortical area contains a retinotopic map, with boundaries between areas characterized by color reversals. Individual areas were identified by overlaying a published map of higher visual areas in the mouse (Marshel et al. 2011) on the retinotopic maps and on a live view of the cortical surface (right). Green boxes indicate approximate fields of view for two-photon imaging across animals. Scale bars, 1 mm. Compass shows posterior–anterior and medial-lateral axis.

Prior to cranial window surgery, animals were administered with the antibiotic Baytril (5 mg/kg, s.c.) and the anti-inflammatory drugs Rimadyl (5 mg/kg, s.c.) and Dexamethasone (0.15 mg/Kg, i.m.). Anesthesia was induced at a concentration of 4% isoflurane in oxygen, and maintained at 1.5–2% for the duration of the surgery. Once fully anesthetized animals were secured in a stereotaxic frame (David Kopf Instruments, Tujunga, CA, USA) and the scalp and periosteum were removed from the dorsal surface of the skull. A custom head plate (see Fig. 1B; Barkus et al. 2022) was attached to the cranium using dental cement (Super Bond, C&B), with an aperture centered over the right hemisphere visual cortex. A 3-mm circular craniotomy was then made, the position of which depended on which visual area was being targeted for imaging. The craniotomy and head plate position was centered −3.1 mm posterior and 2.3 mm lateral from bregma of the right hemisphere; it was closed with a glass insert made from three layers of circular glass (0.15-mm thickness; 1 × 5 mm^2^, 2 × 3 mm^2^ diameter) bonded together with optical adhesive (Norland Products; catalog no. 7106). The window was placed such that the 3-mm circular glass was in contact with the brain surface and the 5-mm-diameter glass rested on the skull surrounding the craniotomy. The window was then sealed with dental cement. After surgery, animals were allowed 2 weeks to recover before being imaged.

### Intrinsic signal imaging

Mice previously implanted with a cranial window and head-plate were placed under anesthesia using isoflurane (5%) and oxygen (0.2 L/min). They were transferred to a heating pad to maintain body temperature at 37°C, and the head-plate was attached to a custom-made holder. Isoflurane was subsequently reduced to 1% for the maintenance of anesthesia throughout the imaging session.

To identify V1 and higher visual areas, a periodic stimulus protocol was used (Kalatsky and Stryker 2003). Computer controlled shutters were used to present stimuli to one eye at a time. These were generated by VSG5 (Cambridge Research Systems) and consisted of a bar of 40° in length and 4° width drifting temporally upwards at a rate of 0.125 Hz and a speed of 15°/second, presented on a cathode ray tube screen positioned in front of the mouse at a distance of 14 cm such that stimuli could be presented in the binocular and the left monocular visual field of the mouse (contralateral to the imaged right hemisphere). The stimulus was presented to each eye six times.

The cortex was illuminated using a halogen light source with interchangeable bandpass filters. Initially green light (546 nm) was used to acquire an image of the cortical surface vasculature. Intrinsic signal imaging (Imager 3001, Optical Imaging Inc, Israel) was carried out using an Adimec 50-R64 camera and running VDAQ software. The cortex was illuminated with red light (700 ± 10 nm). The focal point of the camera was adjusted to 150–200 μm below the surface of the cortex. For each pixel in the imaged region, phase and amplitude of the optical signal at 0.125 Hz were calculated by Fast Fourier Transform (Kalatsky and Stryker 2003). Response amplitudes represent Δ*R*/*R* values (where *R* is light-reflected), and phases represent retinotopic positions of maximal response (which is color coded). The binocular zone of V1 was determined by thresholding the ipsilateral eye response map at 60% of the maximum pixel value.

### Two-photon imaging

In vivo two-photon imaging was performed on awake mice using a resonant scanning microscope (Thorlabs, B-Scope) with a 16× 0.8NA water-immersion objective (Nikon). GCaMP6f was excited at 920 nm using a Ti:sapphire laser (Chameleon, Coherent) with a maximum laser power at sample of 50 mW. Data were acquired at approximately 60 Hz and averaged, resulting in a frame rate of approximately 10 Hz. For all imaging experiments, animals were head-fixed (following prior habituation) and placed on a custom designed cylindrical treadmill whose axis was clamped. Imaging and visual stimulation timing data were acquired using custom written DAQ code (Matlab) and a DAQ card (NI PCIe-6323, National Instruments). Visual stimuli were generated in Matlab using the psychophysics toolbox (Brainard 1997), and displayed on a calibrated LCD screen (Iiyama, B2080HS; width × height 26 × 47 cm^2^) placed 20 cm from the eyes in the binocular visual field of the animal. All visual stimuli were, unidirectionally drifting square wave gratings shown in a circular aperture of 40° diameter, spatial frequency (0.3 cycles per degree) and temporal frequency (1.25 Hz) presented pseudo-randomly at four orientations of 0°, 45°, 90°, and 135° degrees and at full contrast. Drifting gratings were presented for 2 seconds followed by a 5-second inter-trial interval during which a gray screen was displayed. Stimuli were shown to one eye at a time using computer-controlled shutters. In order to determine OD, stimuli were repeated 10 times in a pseudo-random order.

Recordings were made from a 400 × 400 μm (256 × 256 pixels) field of view, at a depth of 150–250 μm (corresponding to layer 2/3), with the position for each HVA chosen on the basis of the retinotopic maps obtained with intrinsic signal imaging and comparison with published area maps of the mouse visual cortex (Wang and Burkhalter 2007; Marshel et al. 2011); an example is shown in Fig. 1. Approximate positions for the four imaged areas were as follows: V1b, −3.4 mm posterior and 2.3 mm lateral from bregma; LM, −3.3 posterior, 3.8 lateral; AL −2.6 mm posterior, 3.5 mm lateral, PM −3.0 posterior, 1.6 lateral. The field of view was identical between recording sessions for each animal, and the region of interest chosen for each HVA was fixed within that field of view. Within the field of view all identifiable neurons were analyzed, but they were not tracked individually across recording sessions.

### Calcium imaging data analysis

Calcium imaging data were registered and segregated into neuronal regions of interest using Suite2P (Pachitariu et al. 2017). Pixel-wise stimulus preference maps were constructed by first calculating the mean of the registered imaging frames recorded during the drifting phase of each stimulus, and then determining for each pixel the stimulus that elicited the largest mean response. Motion correction was applied after frame averaging.

### Quantification and statistical analysis of visual responses

The visual responses of individual neurons were quantified as the mean dF/F value between 0.5–1.5 seconds after stimulus onset, with a baseline period quantified for each trial as the mean dF/F between 1 and 0 seconds before stimulus onset. Orientation tuning curves were fit to a 1d Gaussian function, constrained to peak at the preferred orientation, and with amplitude determined by the response amplitude at this orientation. Fitting was performed using the *fit* function in Matlab. Orientation tuning fit curves were used to calculate an orientation selectivity index OSI defined as OSI = 1 − circular variance, with circular variance calculated by the method of Batschelet (1981).

An ocular dominance index (ODI) was calculated for each neuron as $\boldsymbol{ODI}=\frac{{\boldsymbol{R}}_{\boldsymbol{max}}\boldsymbol{C}-{\boldsymbol{R}}_{\boldsymbol{max}}\boldsymbol{I}}{{\boldsymbol{R}}_{\boldsymbol{max}}\boldsymbol{C}+{\boldsymbol{R}}_{\boldsymbol{max}}\boldsymbol{I}.}$

A neuron was classified as visually responsive if it showed dF/F during visual stimulation being significantly greater (at *P* < 0.01) for at least one stimulus than for baseline, with statistical significance calculated by the Kruskal–Wallis test between the baseline and visual stimulation periods. Kruskal–Wallis tests to measure differences in response amplitude by stimulus orientation were used to assess whether neurons discriminated orientation for stimuli shown to each eye.

For each area of interest, significant changes in the proportions of visually responsive cells out of the total number of recorded cells over the course of the experiment were tested for by use of a continuity-corrected version of Wilson’s test of equal proportions (Wilson 1927) using the prop.test function of the stat package in R.

Wilcoxon tests were carried out to test for significant differences in the distribution of ODI values between baseline and immediately-post MD sessions, and then between the post-MD and 2 weeks binocular recovery sessions. Changes in OSI for cells responding to contralateral and ipsilateral eye stimulation, respectively, were tested for in the same manner.

Correction for multiple testing was implemented by use of the conservative Bonferroni method, to minimize our reporting of false-positive results. Where adjusted *P*-values failed to reach significance but the unadjusted values did, we report both values, such that potential false-negative results likely to arise from stringent multiple comparison correction may also be identified.

## Results

**Fig. 2:**
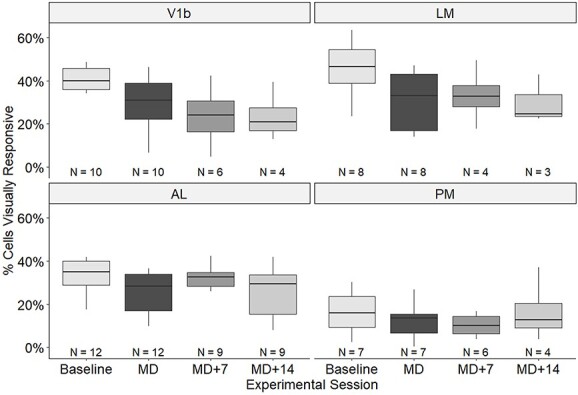
Proportions of visually responsive cells across visual areas and time points. Percentage of cells significantly responsive to visual stimulation out of all cells identified within V1b, LM, AL, and PM across time points. Box plots showing medians and inter-quartile ranges as well as minimum and maximum values. *N*, number of animals for each area and time point.

We obtained V1b data sets consisting of a pre-MD baseline recording, followed by a second recording immediately after 2 weeks of MD from 10 mice, LM data sets from 8 mice, AL recordings from 12, and PM recordings from 7 animals. We were able to further record from a subset of animals after 7d and after 14d binocular recovery (for animal numbers, see Fig. 2). A timeline of the experiments is shown in Supplementary Material, Fig. 1. All calcium imaging data were obtained from awake mice. The fraction of visually responsive neurons for all areas and time points is shown in Fig. 2. In the baseline condition between a third and half of neurons in V1b, LM, and AL were visually responsive according to our criteria (41.5, 44.9, and 36.2%, respectively), but only 13.7% in PM—our visual stimulus parameters were possibly not optimal for this area. The percentage of visually responsive neurons varied over time significantly in all areas (Fig. 2), as a result of MD and binocular recovery (Wilson test for equality of proportions; V1b: *P* = 2.14 × 10^−54^, χ^2^ = 260.2; LM: *P* = 1.75 × 10^−22^, χ^2^ = 112.4; AL: *P* = 1.56 × 10^−19^, χ^2^ = 98.6; PM: *P* = 0.0111, χ^2^ = 19.52.)

### Ocular dominance

**Fig. 3:**
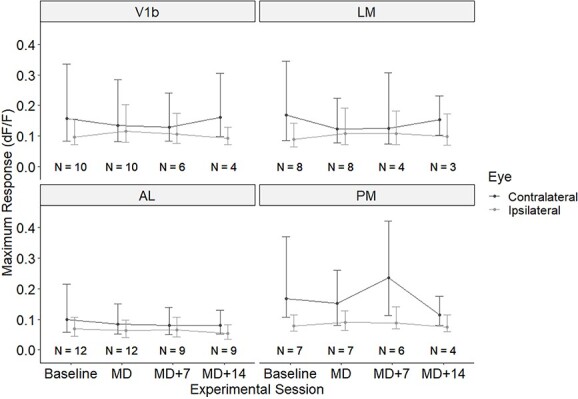
Amplitude of responses through the contra- and ipsilateral eyes of all visually responsive neurons recorded in areas V1b, LM, AL, and PM. Medians and inter-quartile ranges for all visually responsive cells are shown; contralateral eye, black lines, ipsilateral eye, gray lines. *N*, number of animals for each area and time point.

In order to assess the effects of MD and subsequent binocular recovery on the neuronal responses through each eye we calculated the median of the fitted Rmax for all visually responsive neurons separately for contra- and ipsilateral eye stimulation for all four areas and time points; results are shown in Fig. 3. We then performed Wilcoxon tests on the comparisons of (i) baseline vs. immediately post-MD, and (ii) immediately post-MD vs. 2 weeks of binocular recovery.

In areas V1b, LM, and AL, the contralateral eye responses decreased between the baseline recording and the recording immediately post-MD, but this decrease was significant only for AL (from a median of 0.099 to 0.0847; χ^2^ = 862,877, adjusted *P* = 6.14 × 10^−6^) and for LM (from 0.169 to 0.123; χ^2^ = 451,465, adjusted *P* = 2.76 × 10^−5^). The decrease in V1b (from 0.157 to 0.135) was not quite significant (χ^2^ = 872,130, unadjusted *P* = 0.0878); the same applied to PM (decrease from 0.167 to 0.152, χ^2^ = 22,091, unadjusted *P* = 0.0737). Conversely, the ipsilateral eye responses increased significantly in both areas V1b (from a median 0.0972 to 0.115) and LM (from 0.0895 to 0.109), and these increases were highly significant (V1b: χ^2^ = 721,397, adjusted *P* = 2.25 × 10^−5^; LM: χ^2^ = 334,064, adjusted *P* = 9.06 × 10^−5^). There was a smaller, not significant increase of the ipsilateral eye response in PM (from a median 0.0779 to 0.0898; χ^2^ = 17,746, unadjusted *P* = 0.0916). A small decrease of the ipsilateral eye response in AL was also not significant once correction for multiple comparison had been made (from 0.689 to 0.637; χ^2^ = 818,241, unadjusted *P* = 0.00332, adjusted *P* = 0.172).

Following 2 weeks of binocular recovery increases in response through the contralateral (formerly deprived) eye were observed in areas V1b and LM, whereas responses decreased further in both AL and PM; however, none of these changes were significant even before corrections for multiple comparisons had been made. In contrast, responses through the ipsilateral eye exhibited decreases over the same period of time in all four areas. These were significant in V1b (χ^2^ = 67,400, adjusted *P* = 1.87 × 10^−5^) and AL (χ^2^ = 276,938, adjusted *P* = 0.00443) but not in LM (χ^2^ = 35,867, unadjusted *P* = 0.284) and PM (χ^2^ = 4027, unadjusted *P* = 0.226).

We classified visually responsive neurons as contralateral if the response to contralateral-eye stimulation was at least five times as high as the response to ipsilateral-eye stimulation; the converse applied to neurons classified as ipsilateral. All neurons for which responses through one eye were less than five times as high as those through the other eye were classified as binocular. In line with previous studies (Gordon and Stryker 1996), we found that at least 75% in all four areas were binocular by that definition, between 15 and 20%, were contralateral and around 5% were ipsilateral (Supplementary Material, Fig. 3). MD caused a decrease in the proportion of contralateral cells in all areas as well as an increase in binocular cells, with little change in the proportion of ipsilateral neurons, but this classification proved too insensitive for a more quantitative analysis.

**Fig. 4:**
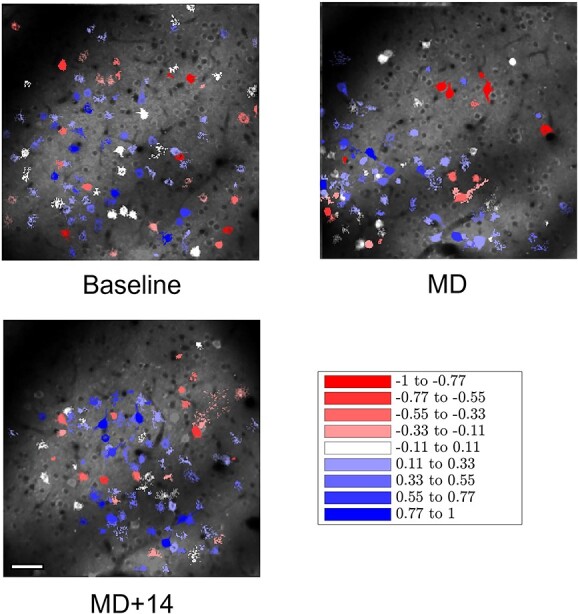
Changes to the ODI over time of all visually responsive cells in area AL of one mouse. All visually responsive cells in the baseline recording (top left) were color coded after dividing the population into nine equal bands ranging from ODI = −1.0 (red) to 1.0 (blue). The same ODI bands were then used to color code the distribution of visually responsive cells following MD (top right) and after 14 days of binocular recovery (bottom left). Scale bar, 50 μm.

**Fig. 5:**
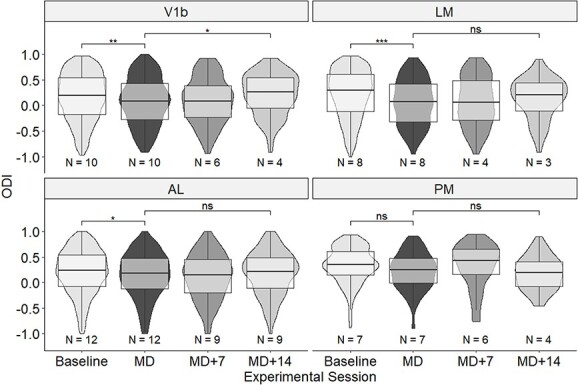
Changes to ODI caused by MD and binocular recovery. ODI for visual areas V1b, LM, AL, and PM across time points. Violin plots show medians and interquartile ranges, minimum and maximum values as well as density of ODI values of individual neurons. Note that these have been normalized for the overall number of neurons imaged for each time point and cortical area. *N*, number of animals for each area and time point. Significant differences are indicated by ^*^P < 0.05, ^*^^*^P < 0.01, and ^*^^*^^*^P < 0.001.

Effects of MD and recovery after reopening of the deprived eye are most commonly assessed through the ODI although this measure does not reveal the underlying changes in response through the individual eyes (see above). An example of the changes seen for all visually responsive cells identified in area AL of one animal over time is shown in Fig. 4. Population data for all areas across time are shown in Fig. 5.

As expected, in V1b, there was a significant shift in ODI from 0.195 to 0.084 during 2 weeks of MD (Wilcoxon, *P* = 0.00443, *W* = 921,661). During 2 weeks of recovery there was a significant shift back to an ODI of 0.256 (Wilcoxon, *P* = 0.0179, χ^2^ = 46,517). In comparison, in the ventral-stream area LM we observed a more pronounced shift in ODI from 0.295 to 0.0746 (Wilcoxon, *P* = 2.00 × 10^−9^, *W* = 470,585), and a partial recovery to 0.209 during the subsequent 2 weeks of binocular vision, although this lost significance following correction for multiple comparisons (Wilcoxon, unadjusted *P* = 0.0477, adjusted *P* = 0.999, *W =* 30,253).

In AL, the decrease in ODI (from 0.237 to 0.179) during 2 weeks of MD was more modest but still significant after correction for multiple comparison (Wilcoxon, *P* = 0.0276, *W* = 826,338); the ODI then decreased further to 0.149 after 1 week of binocular vision before returning to near baseline after 2 weeks (Wilcoxon, unadjusted *P* = 0.148, *W* = 235,673). In PM, the ODI also decreased during MD, from 0.351 to 0.251, but this was not quite significant after correction for multiple comparison (Wilcoxon, unadjusted *P* = 0.00117, adjusted *P* = 0.0611, *W* = 23,911). After 1 week of recovery, the ODI increased to 0.430 but then decreased again, to 0.193 after 2 weeks of binocular vision; this shift was not significant (Wilcoxon, unadjusted *P* = 0.455, *W* = 3875).

**Fig. 6:**
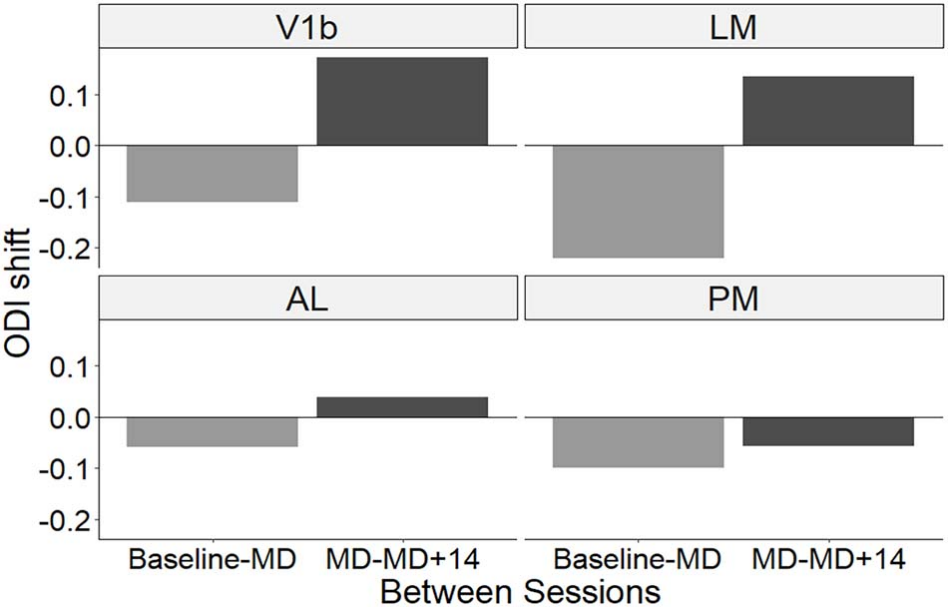
ODI shifts during 2 weeks of MD and during 2 weeks of binocular recovery for visual areas V1b, LM, AL, and PM. For each area, the difference between the median ODI at baseline and at the end of the MD period (gray bars) and the difference between the median ODI at the end of the MD period and after 2 weeks of binocular recovery (back bars) is plotted.

In summary, a very strong and significant shift in ODI was seen in the ventral steam area LM following MD; the OD shift in V1b was smaller, and those in dorsal-steam areas AL and PM were smaller still. Within 2 weeks of restored binocular vision, a full or partial recovery of the ODI was observed in V1b, LM, and AL, but not in PM (Fig. 6).

### Orientation selectivity

**Fig. 7:**
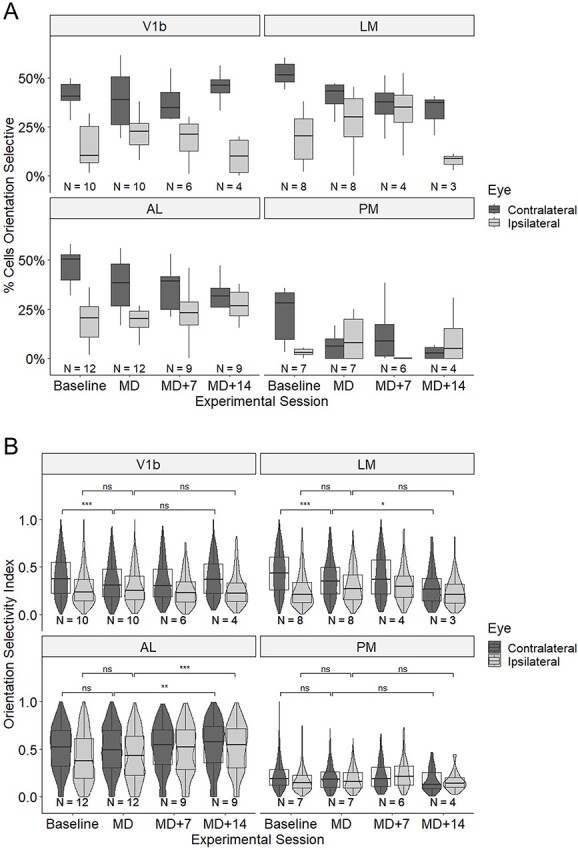
Effects of MD and binocular recovery on orientation selectivity of visually responsive neurons in cortical areas V1b, LM, AL, and PM. (A) Percentage of orientation selective cells responding to contralateral and ipsilateral eye stimulation, respectively, before and after MD and following binocular recovery. Box plots showing medians and interquartile ranges as well as minimum and maximum values. (B) Orientation selectivity index (OSI) of cells responding to contralateral and ipsilateral eye stimulation, respectively, before and after MD and following binocular recovery. Violin plots showing medians and interquartile ranges, minimum and maximum values as well as density of OSI values of individual neurons. *N*, number of animals for each area and time point. Significant differences are indicated by ^*^P < 0.05, ^*^^*^P < 0.01, and ^*^^*^^*^P < 0.001.

It is worth noting that the percentage of orientation selective cells out of all cells responding through the contralateral eye was above 40% not only in area V1b (42.8%), but also in LM (54.0%) and AL (47.7%), whereas it was only 22.0% in area PM (Fig. 7A).

In order to quantify potential effects of MD on orientation selectivity, we calculated the OSI of all visually responsive neurons to contra- and ipsilaterally presented gratings (Fig. 7B). Comparing responses immediately after reopening of the deprived (contralateral) eye with baseline responses 2 weeks earlier, we observed a highly significant reduction in OSI for the contralateral eye in V1b, from a median of 0.374 to 0.306 (Wilcoxon test, *W* = 957,534, *P* = 1.59 × 10^−6^). There was a corresponding increase in OSI of responses through the ipsilateral eye, from a median of 0.233 to 0.252, but this failed to reach significance once correction was made for multiple comparisons (Wilcoxon test, *W* = 774,503, unadjusted *P* = 0.00913, adjusted *P* = 0.475). Area LM also exhibited a reduction in OSI of contralateral (deprived) eye responses, from a median of 0.428 to 0.343, but this was not significant after correction for multiple comparisons (*W* = 360,775, unadjusted *P* = 0.00535, adjusted *P* = 0.278). There was again a corresponding increase in the OSI of ipsilateral-eye responses, from a median of 0.219 to 0.249; however, this increase did not reach significance once correction was made for multiple comparisons (*W* = 273,002, unadjusted *P* = 0.00432, adjusted *P* = 0.225). For areas AL and PM, we also observed small decreases in OSI for contralateral eye stimulation and small increases for ipsilateral eye stimulation, but none of these changes were significant.

Following 2 weeks of binocular recovery, in V1b, the OSI of responses through the contralateral eye returned to near baseline (median, 0.371), but the increase failed to reach significance once correction for multiple comparison had been made (Wilcoxon test, *W* = 48,627, unadjusted *P* = 0.00535, adjusted *P* = 0.273). Conversely, the OSI of responses through the ipsilateral eye decreased to near baseline (median, 0.224); however, this decrease did not quite reach significance (*W* = 60,795, unadjusted *P* = 0.0665). Surprisingly, we observed a significant increase in the OSI of both contra- and ipsilateral eye responses in AM: for contralateral-eye stimulation the median OSI increased from 0.494 to 0.579 and for ipsilateral-eye stimulation from 0.434 to 0.550 (*P* = 0.00843 and 4.34 × 10^−8^, respectively). There was no recovery of the OSI for contralateral responses during 2 weeks of binocular vision for either LM or PM, in fact for both areas the OSI decreased further.

## Discussion

The principal findings of our study are summarized in Table 1, in terms of the effect of MD and recovery on the absolute strength of responses through the two eyes, OD, and orientation selectivity.

**Table 1:** Summary of main findings. Bold arrows indicate significant changes, thin arrows changes that lost significance after correction for multiple comparisons, and ↔ indicate non-significant changes. Rmax, median maximal response: NDE, non-deprived (ipsilateral) eye; DE, deprived (contralateral) eye.

	14d MD		14d recovery		14d MD	14d recovery	14d MD		14d recovery	
	Rmax DE	Rmax NDE	Rmax DE	Rmax NDE	ODI	ODI	OSI DE	OSI NDE	OSI DE	OSI NDE
V1b	↔		↔					↑	↑	↔
LM			↔	↔		↑	↓	↑	↓	↔
AL		↓	↔			↔	↔	↔		
PM	↔	↔	↔	↔	↓	↓	↔	↔	↔	↔

The effects of MD on visual responses in V1b are characterized by a shift in ODI toward the open eye, a decrease in response strength (which was not quite significant) and in orientation selectivity through the deprived eye and an increase in non-deprived eye responses. Although our study was carried out on young adult mice, the MD effects on deprived eye response are, in LM and to some extent in V1b, similar to those previously reported for MD during the critical period (Gordon and Stryker 1996; Frenkel and Bear 2004), which have been attributed to long-term depression of deprived eye inputs (Yoon et al. 2009). In contrast, MD in older mice has more commonly been reported to cause an NMDA receptor dependent upregulation of responses through the non-deprived eye (Sawtell et al. 2003; Ranson et al. 2012). However, under conditions of environmental enrichment adult OD plasticity more closely resembles critical period plasticity in that MD causes a reduction of deprived eye responses (Greifzu et al. 2014). Social experience also enhances OD plasticity (Balog et al. 2014). Our mice, which were group housed and experienced enrichment in form of nesting material and tubes in their cages, showed enhanced V1 plasticity with respect to deprived-eye responses when compared with adult animals housed under so-called standard conditions.

Both the amplitude of responses in V1b and their orientation selectivity largely recovered during a subsequent period of concordant binocular vision. The overall pattern of effects of MD and recovery was similar in area LM (considered part of the ventral stream), as was the decrease in orientation selectivity through the deprived eye. In contrast, area AL and area PM (in the dorsal stream) exhibited smaller changes in the response through the deprived eye, the overall OD, and the orientation selectivity of responses.

There are surprisingly few reports on experience dependent, and more specifically OD, plasticity in higher visual cortical areas from any mammalian species. OD plasticity is widely viewed as a consequence of the convergence of retinal inputs representing the two eyes and a prerequisite for the precise matching of the tuning of response to stimulation of the two eyes (Wang et al. 2010; Gu and Cang 2016; Chang et al. 2020; Tan et al. 2020). If this is the case, then one might expect OD plasticity to occur principally in neurons in the primary visual cortex since eye-of-origin information is largely lost beyond it in all species studied so far, and to be subsequently “inherited” by the higher visual areas that receive input from V1. If, on the other hand, OD plasticity is linked to the development of disparity sensitivity, then one might expect to observe it in higher visual areas where such selectivity is present. Outside of V1, disparity tuning of neurons has been reported for areas LM and RL, which are the areas with the largest representation of the binocular visual field in mice (Garrett et al. 2014; Zhuang et al. 2017). Area RL is tuned mainly to near disparities, representing visual stimuli very close to the mouse (La Chioma et al. 2019), whereas area LM (but not RL) exhibits true stereo sensitivity, with neurons responding more strongly to correlated than to anti-correlated random-dot correlograms (La Chioma et al. 2020). Such responses are typical of visual areas of the ventral stream in primates, namely V4 and inferotemporal cortex. It is therefore interesting to note that LM exhibited a significant OD shift in response to MD, of greater magnitude than that observed in V1. This finding is paralleled by similar results from cat area 21a, considered the gateway to the ventral stream in that species, where OD plasticity has also been found to be stronger (and more long-lasting) than in V1 (Wang et al. 2019).

The fact that the OD shift following MD is greater in ventral-stream area LM than in V1b (although it is smaller in the dorsal stream areas AL and PM) suggests that changes in OD in higher visual areas are not uniformly inherited from V1b. It appears likely that inter-areal differences in plasticity reflect different degrees in plasticity of subpopulations of V1 neurons projecting to those areas. It is well established that visual response characteristics such as spatial and temporal frequency selectivity of neurons in mouse HVAs tend to match those of V1 neurons projecting to those areas (Glickfeld et al. 2013; Matsui and Ohki 2013). Although this is true for areas LM and AL it is not for PM (Murgas et al. 2020), which has tuning characteristics that are atypical of the dorsal stream within which it is placed by most authors (Murakami et al. 2017).

For primates, it has long been known that the severity of amblyopia as assessed by tests of visual acuity cannot be (fully) explained by the neuronal response deficits in the primary visual cortex (Kiorpes et al. 1998; Kiorpes and McKee 1999), suggesting that responses in higher visual areas are more strongly affected. Similarly, in cats, reopening of a previously deprived eye leads to only a partial recovery of visual acuity in that eye despite a full restoration of balanced OD and normal orientation selectivity (Kind et al. 2002), indicating persisting deficits in higher visual areas. The relationship between basic visual functions such as visual acuity and neuronal response properties in higher visual cortical areas requires further study across species.

## Supplementary Material

Plasticity_of_higher_visual_cortical_areas_in_the_mouse-supplementary_bhad203

## Data Availability

Data are available from the corresponding author upon request.
