# Supplementary material for: Experience dependent plasticity of higher visual cortical areas in the mouse
Source: Cereb Cortex. 2023 Jun 3;33(15):9303–12. doi: 10.1093/cercor/bhad203 (PMC10393491; doi:10.1093/cercor/bhad203)
Supplement: Plasticity_of_higher_visual_cortical_areas_in_the_mouse-supplementary_bhad203 [file plasticity_of_higher_visual_cortical_areas_in_the_mouse-supplementary_bhad203.pdf]

Experience dependent plasticity of higher visual cortical areas in the mouse

Rosie Craddock, Asta Vasalauskaite, Adam Ranson, Frank Sengpiel

Supplementary Material

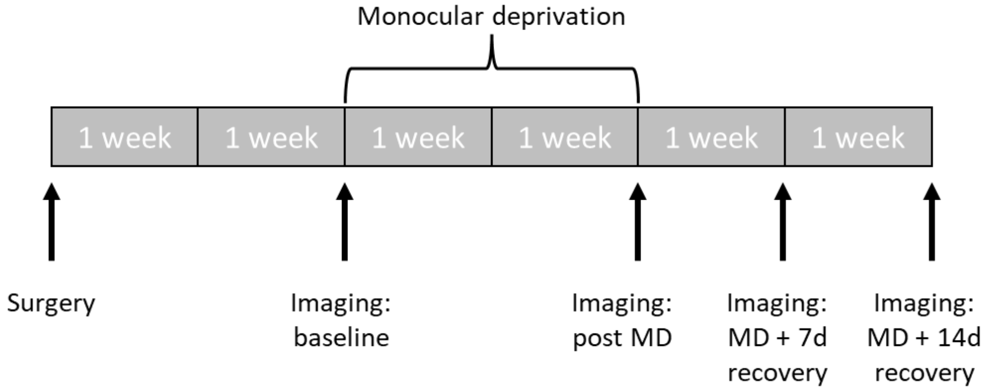

Supplementary Figure 1

Suppl. Fig. 1: Timeline of experiments

Two weeks after cranial window surgery baseline two photon imaging of right visual cortex was carried out, followed by monocular deprivation of the left eye. Two weeks later, the deprived eye was re-opened, and the same area of visual cortex was imaged a second time. One and two weeks later, a third and fourth imaging session were carried out.

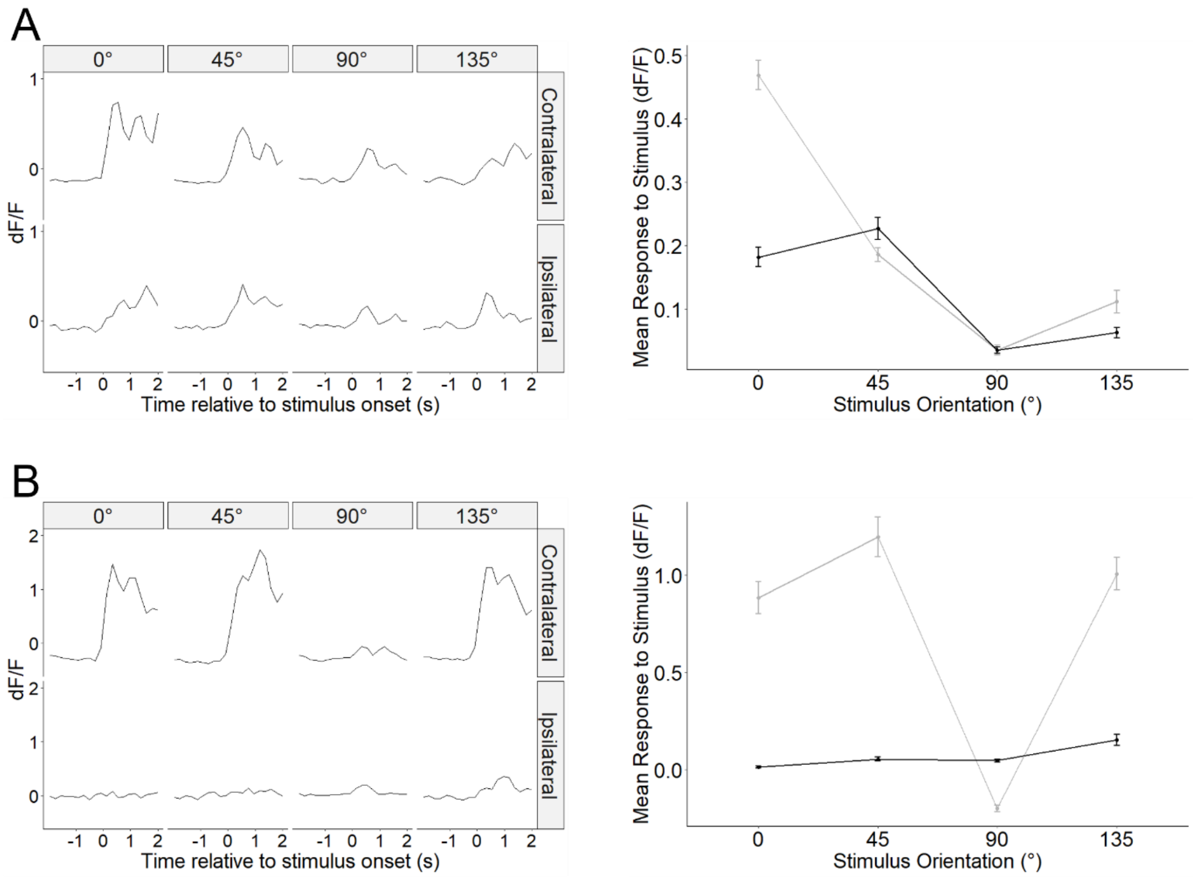

Supplementary Figure 2

Suppl. Fig. 2: Calcium signals and orientation tuning curves of individual neurons.

(A) and (B) show responses of two neurons recorded from area AL; panels on the left show calcium signals (averaged over 10 trials) in response to gratings of 4 orientations shown to the contralateral or ipsilateral eye (for details, see Methods). Panels on the right show orientation tuning curves for responses through the contralateral eye (grey line) and ipsilateral eye (black line), respectively (mean ± sem).

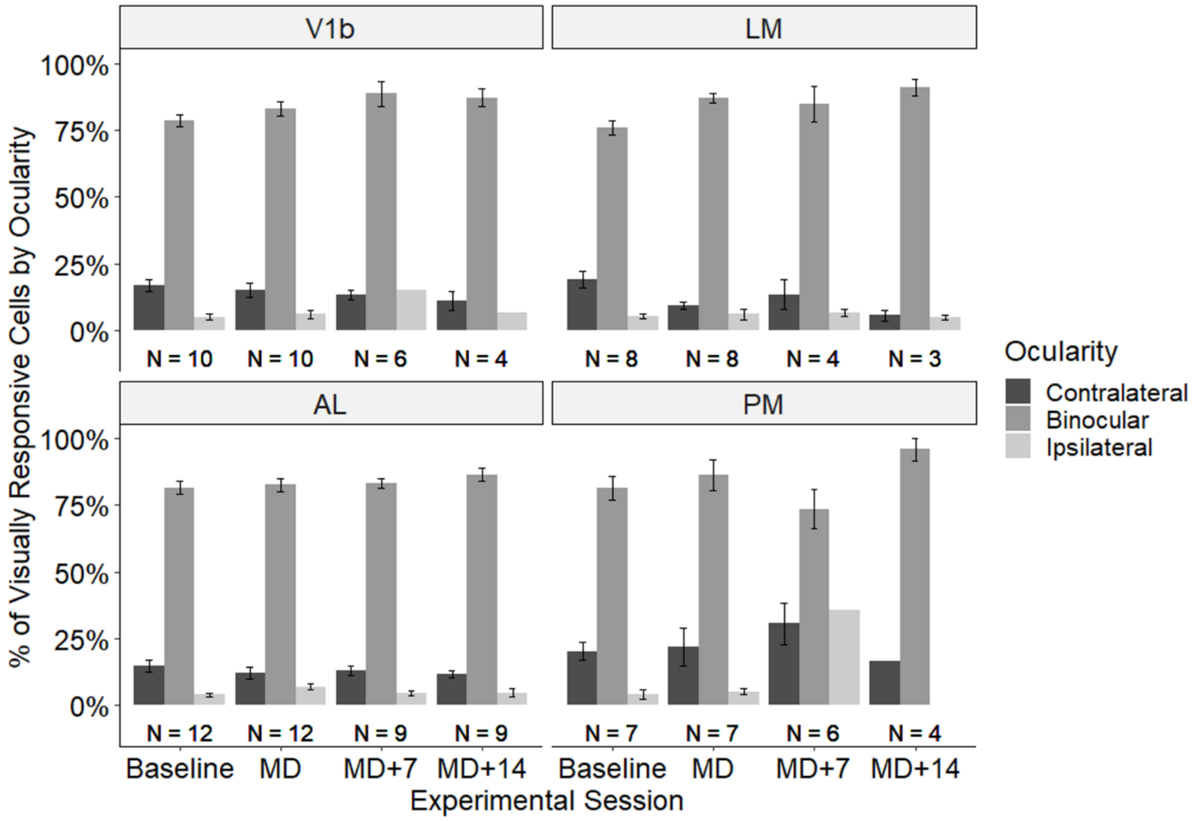

Supplementary Figure 3

Suppl. Fig. 3: Ocularity of all visually responsive neurons recorded in areas V1b, LM, AL and PM .

Visually responsive neurons were categorised as contralateral (black bars), binocular (dark grey bars) or ipsilateral (light grey bars) according to the ratio of their responses to contra- and ipsilateral eye stimulation (for details, see text.
